# 

**Published:** 1894-09-22

**Authors:** 


### SPECIAL EDUCATIONAL NUMBER.

### CONTENTS.

**The International Congress at Buda Pesth** - - - - 493

**Around the Hospitals** - - - - - - - - - 494

**Student Life in the Sixteenth Century** - - - - 495

**Modern Medico-Psychology and Psychiatry.**—By T. S. Clouston, M.D. XI.—Conditions of Mental Exaltation and Excitement - - - - - - - - - - - - 496

**Notes and News** - - - - - - - - - - - - 498

**Annotations.**—A New Cure: The Ass to the Rescue—Undignified French Dignities—The Asylum of the World - - - - - 497

**The Medical Student's Vade Mecum:**

The Metropolitan Medical Schools.—The London Hospital College—Guy's—St. Bartholomew's—St Thomas's—University College—King's College—Middlesex—St. George's—St. Mary's—Charing Cross—Westminster—The School of Medicine for Women—Fees - - - - - - - - - - - 499

### EXTRA SUPPLEMENT.—THE NURSING MIRROR.

**News from the Nursing World.**—Royal National Pension Fund for Nurses—Our Christmas Competitions—Epileptic Colonies—A Nurse-Heroine—Unavoidable Postponement—The Ward Thermometer—Paupers at Portsea—Queen's Nurses at Perth—Nursing at Newtownards—Training of Midwives in India—Justice to Children—Educated Nurses—Lepers in South Africa—After the Event—Short Items - ccxxxix
**Diet in Disease.**—By Mrs. Ernest Hart. III.—Consumption - - - - - - - - - - - ccxli
**Irish Hospitals.**—The Royal Hospital for Incurables - - ccxlii
**Where to Go** - - - - - - - - - - - ccxlii
**Novelties for Nurses** - - - - - - - - - ccxlii
**Presentation** - - - - - - - - - - - ccxlii
**Structure and Care of the Teeth.**—IV.—Children's Teeth Troubles - - - - - - - - - ccxliii
**Women's Work.**—Medical Women - - - - - - ccxliv
**Appointments—A Case for Assistance** - - - - ccxliv
**Minor Appointments—Notes and Queries** - - - - ccxliv

